# GPIS-Based Calibration for Non-Overlapping Dual-LiDAR Systems Using a 2.5D Calibration Framework

**DOI:** 10.3390/s26030800

**Published:** 2026-01-25

**Authors:** Huan Yu, Xiaohong Zhang, Ming Li, Desheng Zhuo, Pin Zhang, Man Li, Yuanyuan Shi

**Affiliations:** 1 School of Geodesy and Geomatics, Wuhan University, Wuhan 430079, China; yuhuanzjj@whu.edu.cn (H.Y.); zhuods@whu.edu.cn (D.Z.); 2 Chinese Antarctic Center of Surveying and Mapping, Wuhan University, Wuhan 430079, China; 3 Hubei Luojia Laboratory, Wuhan 430079, China; 4 School of Artificial Intelligence, Wuhan University, Wuhan 430072, China; liming751218@whu.edu.cn; 5 School of Computer Science, Wuhan University, Wuhan 430079, China; pzhang1992@whu.edu.cn; 6 Dongfeng Motor Corporation Technical Center, Wuhan 430010, China; sunjay.lee@outlook.com (M.L.); minyshi@163.com (Y.S.)

**Keywords:** dual-LiDAR systems, extrinsic calibration, GPIS, surface modeling, non-overlapping field of view, autonomous driving

## Abstract

Dual-LiDAR systems are widely deployed in autonomous driving, yet extrinsic calibration remains challenging in non-overlapping field-of-view (FoV) configurations where correspondence-based methods are unreliable. We propose an engineering-oriented 2.5D calibration framework that estimates horizontal extrinsics (x,y,yaw) via motion-guided planar alignment and then refines them using Gaussian Process Implicit Surfaces (GPIS), which provide continuous and probabilistic surface constraints from spatially disjoint scans. This design avoids calibration targets and reduces dependence on strong scene assumptions, improving robustness under noise and weak structure. Extensive high-fidelity simulation experiments demonstrate centimeter-level lateral accuracy and sub-degree yaw error, consistently outperforming representative motion-based and BEV-based baselines under both clean and noisy settings. To further assess real-world applicability, we conduct a preliminary nuScenes case study by splitting LiDAR scans into front and rear subsets to emulate a non-overlapping dual-LiDAR setup, achieving improved yaw accuracy and competitive lateral precision. Overall, the proposed method serves as a practical refinement stage for non-overlapping dual-LiDAR calibration, with a favorable balance of accuracy, robustness, and engineering feasibility.

## 1. Introduction

The adoption of multi-LiDAR configurations has become increasingly prevalent in autonomous driving systems. Accurate extrinsic calibration, particularly in non-overlapping sensor layouts, is critical to achieving reliable sensor fusion and high-quality environmental perception.

Recent surveys [1] confirm that existing calibration methods struggle with minimal overlap scenarios. While prior works—such as motion-based calibration pipelines with geometric refinement [2] or coarse-to-fine strategies leveraging road structures [3]—have shown promising results in structured scenes, their performance often deteriorates in unstructured environments. This degradation is especially pronounced in non-overlapping LiDAR setups, where minimal FoV overlap undermines conventional point- or plane-based registration methods. While some approaches [4,5] have addressed non-overlapping configurations, they typically require strong environmental priors or specific sensor arrangements. Recent benchmarks such as BLCC [6] have further established standardized evaluation metrics for multi-LiDAR systems, highlighting the need for robust, targetless solutions.

Several practical challenges contribute to these limitations. First, heterogeneous sensor configurations—such as variations in vertical angular resolution (0.1° to 1.33°) and horizontal resolution (0.05° to 0.4°) [7]—complicate direct alignment between LiDAR devices. Second, due to physical installation constraints, FoV overlap between sensors is often below 15% [2,8], rendering conventional feature correspondence unreliable. Moreover, vehicle-mounted LiDARs frequently exhibit anisotropic angular sampling, amplifying vertical misalignment. Real-world point clouds also tend to be sparse, noisy, and lack geometric texture, which further challenges explicit surface modeling approaches.

To this end, we propose a novel extrinsic calibration framework based on GPIS for non-overlapping dual-LiDAR sensor setups in autonomous vehicles. By constructing continuous, probabilistic surface models from disjoint LiDAR observations, our method performs reliable and robust calibration even in unstructured environments, without requiring overlapping fields of view or artificial targets. From a sensor-system perspective, the proposed framework is designed as a modular calibration backend that can be integrated into LiDAR-based perception and mapping stacks to improve geometric consistency across sensors. The approach integrates relative motion constraints and GPIS-based surface registration within a hybrid optimization pipeline.

Despite these efforts, existing decoupled strategies frequently neglect formal error propagation under sensor noise or motion uncertainty, reducing their reliability in field applications. Together, these challenges underscore the need for a calibration method that can operate effectively without overlapping views, artificial targets, or strong geometric priors.

The main contributions of this paper are as follows:

- GPIS-based implicit surface modeling for non-overlapping LiDAR calibration: we build a continuous surface representation with a thin-plate spline kernel and a confidence-weighted formulation to handle sparse and noisy LiDAR returns.
- A practical 2.5D calibration pipeline for engineering deployment: we propose a multi-stage framework that estimates horizontal extrinsics (x,y,yaw) via motion/planar alignment and refines them via GPIS surface alignment; vertical-aware preprocessing follows our prior work [8] and is not quantitatively evaluated in this paper.
- Comprehensive evaluation in simulation and a preliminary real-data case study: we validate accuracy and robustness under varying Gaussian noise in PreScan and further report a nuScenes-based case study using front/rear split point clouds to emulate non-overlapping dual-LiDAR setups.

The rest of this paper is organized as follows: Section 2 reviews relevant multi-LiDAR calibration techniques. Section 3 presents the proposed GPIS-based calibration framework. Section 4 reports experimental results and comparisons. Section 5 concludes this paper and outlines future directions.

## 2. Related Work

Accurate extrinsic calibration of multi-LiDAR systems is critical for enabling robust sensor fusion and environment modeling in autonomous platforms. Existing works can be broadly categorized into four main classes: (1) targetless calibration, (2) scene-structure-driven calibration, (3) integrated systems with joint optimization, and (4) implicit surface modeling for calibration. This section reviews representative methods in each category and highlights their limitations in non-overlapping configurations.

### 2.1. Targetless Calibration via Motion or Overlap Constraints

Target-free calibration strategies have been extensively studied for LiDAR systems, with methods leveraging point cloud geometry and motion consistency [9]. These methods aim to estimate inter-LiDAR extrinsics without relying on artificial calibration targets. Some motion-based calibration techniques trace their roots to classical hand–eye calibration theory, including dual quaternion formulations [10], which inspire modern LiDAR-to-LiDAR self-alignment strategies. Jiao et al. [2] proposed a motion-based pipeline combining hand–eye initialization and geometric point-to-plane refinement, which performs well in moderate overlap scenarios. Maroli et al. [11] introduced a rotation-only calibration technique using vehicle motion, but it lacks support for full 6-DoF estimation. Zhu and Liu [12] presented an early unsupervised approach relying on heuristic constraints from LiDAR returns, yet it suffers from limited observability and lacks probabilistic modeling.

Recent studies have extended these ideas to multi-modal configurations. Zhang et al. [4] proposed an overlap-free calibration method for LiDAR–camera platforms by leveraging environmental structure, demonstrating that overlapping FoVs are not strictly necessary for accurate calibration. Liu et al. [5] developed a targetless calibration method using adaptive voxelization to align small FoV LiDARs and cameras, showing promising results under sparse data conditions. Recent developments include Multi-LiCa [13], which employs feature-based matching with GICP refinement, and overlap-free methods [4] that leverage environmental structure. However, these approaches often require partial overlap between sensors or rely on specific environmental features, limiting their applicability in diverse scenarios. Adaptive point-line fusion techniques [14] further enhance robustness in non-overlapping configurations. These strategies reinforce the feasibility of structure-aware and probabilistic approaches in non-overlapping setups and provide valuable inspiration for our GPIS-based method.

### 2.2. Scene-Structure-Driven Calibration

Scene-based methods enhance robustness by leveraging strong environmental priors. Occupancy grids [15] and scan-matching techniques such as Normal Distributions Transform (NDT) [16] have shown utility in structured settings but require sufficient overlap. Wei et al. [3] introduced CROON, a two-stage framework using road plane estimation and ICP-based refinement. M-LOAM [17] integrates calibration within SLAM, jointly optimizing extrinsics and motion. LeGO-LOAM [18] and Loam-Livox [19] provide robust odometry front-ends often reused in calibration pipelines.

Point cloud preprocessing techniques such as fast 3D segmentation [20], planar surface extraction [21], and shape detection with Random Sample Consensus (RANSAC) [22] are also commonly applied. Probabilistic map structures such as OctoMap [23] further improve robustness in complex environments. Despite their effectiveness, these methods generally depend on geometric regularity, dense surfaces, and loop closure, which limits their use in open or weakly structured environments.

### 2.3. Integrated Systems with Joint Optimization

A parallel research direction embeds calibration into SLAM frameworks for joint optimization [24]. M-LOAM [17] jointly estimates trajectory and calibration parameters using a sliding window. Recent unified frameworks such as iKalibr [25] support multiple sensor modalities with continuous-time trajectory estimation, while observability-aware methods [26] analyze parameter identifiability. Continuous-time approaches [27,28] further enhance modeling for asynchronous sensors. While integrated approaches offer adaptive refinement, they are sensitive to odometry quality and may suffer from drift accumulation [17].

In contrast, our method decouples calibration from motion tracking and map building, enabling reliable performance without SLAM or loop closure assumptions.

### 2.4. Implicit Surface Modeling for Calibration

Recent advances in probabilistic modeling have enabled implicit and continuous representations of geometry. GPIS [29] represent signed distance fields, (SDFs) with uncertainty estimates, allowing smooth interpolation across sparse and noisy inputs. Applications of GPIS include robotic grasping [30], semantic SLAM [31], control barrier functions [32], and robotic manipulation [33]. Recent work has extended GPIS to unified frameworks for mapping, odometry, and planning [31]. Extensions such as Log-GPIS distance fields [34], GPmap for sparse inference [35], continuous frontier mapping [36], and GP occupancy mapping [37] improve computational scalability and flexibility.

To the best of our knowledge, our work is the first to employ GPIS as a core constraint mechanism for extrinsic calibration in multi-LiDAR setups. By learning probabilistic surface priors from spatially disjoint scans and embedding them into a hybrid optimization framework, we avoid reliance on overlap, targets, or strong structural assumptions. This enables robust calibration in minimally constrained, unstructured, and noisy environments—conditions where many existing techniques degrade.

Even under non-overlapping instantaneous FoVs, many pipelines implicitly create map-level shared structure by aggregating scans into a common reference frame and then performing alignment (e.g., ICP/NDT/feature matching) on the accumulated representation. However, dense-map registration can be brittle under noise/outliers and often yields a non-smooth objective that is difficult to optimize robustly. We therefore introduce GPIS as an intermediate representation layer: it denoises measurements, provides a continuous implicit surface, and stabilizes linearization during optimization. A qualitative comparison of representative multi-LiDAR calibration paradigms and their limitations under non-overlapping setups is summarized in Table 1. In this work, we benchmark only 2.5D horizontal extrinsics (x,y,yaw), while vertical-aware preprocessing follows our prior work and is therefore not quantitatively evaluated.

## 3. Materials and Methods

### 3.1. System Overview

**Figure 1:**
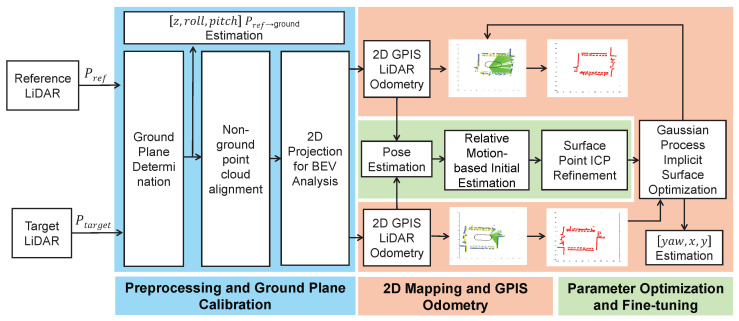
Overview of the proposed extrinsic calibration pipeline. The system consists of three main modules: (1) preprocessing for ground plane estimation and bird’s-eye view (BEV) projection; (2) GPIS-based LiDAR odometry with SDF alignment and continuous mapping; and (3) multi-stage extrinsic calibration including motion-based initialization and surface-level refinement. The front LiDAR is treated as the reference sensor, while the rear-mounted LiDAR is the target.

We propose a novel framework for extrinsic calibration of non-overlapping multi-LiDAR systems, leveraging GPIS to construct continuous surface models and enable high-precision alignment. The framework consists of three main stages, as illustrated in Figure 1:

1. Preprocessing: vertical component estimation and BEV projection to improve surface completeness and provide a structured prior.
2. GPIS-based LiDAR odometry: frame-to-frame motion estimation via SDF alignment and map updates using GP regression.
3. Extrinsic calibration: initialization through motion constraints followed by refinement using ICP and GPIS surface registration.

**Figure 2:**
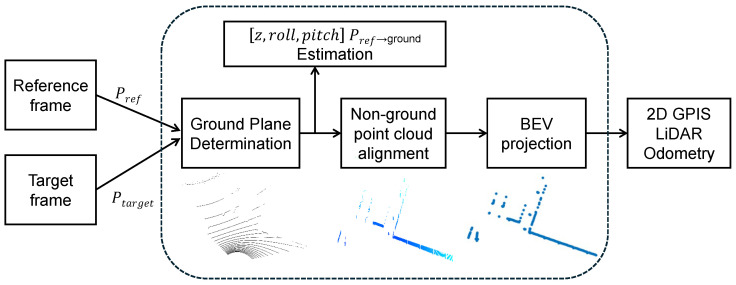
Preprocessing pipeline for GPIS-based odometry. Reference and target LiDAR frames undergo ground plane estimation, non-ground point alignment, and bird’s-eye view (BEV) projection. These structured inputs serve as priors for the subsequent GPIS surface modeling, enhancing robustness under sparse or noisy observations.

Preprocessing: Vertical-Aware Ground Estimation and BEV Projection. To mitigate the sparse vertical resolution in LiDAR scans, we adopt a vertical-aware preprocessing routine to stabilize ground estimation and BEV construction. Enhanced RANSAC fitting and dynamic occupancy encoding yield robust ground estimation. These preprocessing steps, validated in our previous work [8], provide reliable structural priors for downstream GPIS modeling. Note that the quantitative evaluation in this paper focuses on 2.5D horizontal extrinsics (x,y,yaw); vertical-related parameters are leveraged only for preprocessing and are not benchmarked here. The full preprocessing pipeline is illustrated in Figure 2.

GPIS-based Odometry. We employ GPIS to model the observed environment as a SDF. Each new frame’s pose is estimated by minimizing the distance between its points and the SDF surface. The map is updated using GP regression while discarding low-confidence regions, maintaining consistency and smoothness.

Extrinsic Calibration. Initial extrinsic parameters are estimated using relative motion constraints between LiDARs, formulated as a nonlinear hand-eye calibration problem. These parameters are then refined through ICP alignment of GPIS zero-level surfaces, achieving accurate calibration without overlap or targets.

This pipeline decouples mapping from calibration, ensuring modularity and robustness in unstructured, non-overlapping configurations.

### 3.2. LiDAR Odometry via GPIS Mapping

In this work, we introduce a robust method for LiDAR pose estimation and map updating based on GPIS [30,31]. GPIS is a sophisticated mathematical tool that effectively represents the shape of 2D and 3D surfaces in an implicit manner, aligning well with real-world environmental structures.

The essence of our method lies in its ability to predict surfaces directly, eliminating the need for explicit parametric surface representations. This characteristic makes it an ideal solution for handling complex spatial geometry problems. The implicit surface is mathematically defined using the two-dimensional thin plate spline covariance function. This function offers several advantages: it describes smooth surfaces, ensures infinite differentiability, requires minimal manual tuning under our default settings (kernel form fixed; a small number of interpretable hyperparameters), provides a closed-form solution, and has physical significance through its energy equation. Additionally, the overlap range between consecutive frames serves as an effective measure for similarity estimation, reinforcing the GP as an optimal choice for the optimization process. The overall algorithm flow is as follows:

#### 3.2.1. Pose Estimation

**Figure 3:**
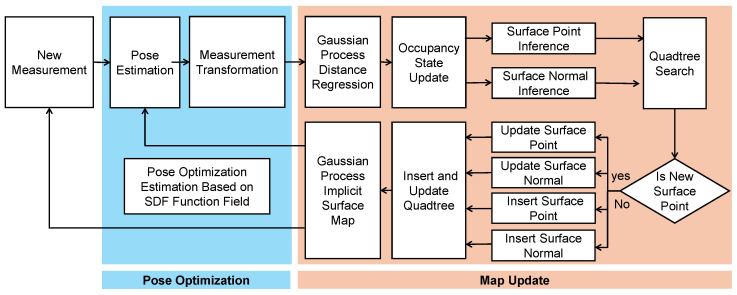
Flowchart of the proposed GPIS-based LiDAR odometry and mapping framework. The system consists of two main modules: (1) pose optimization based on SDF alignment; and (2) surface map updating via Gaussian process (GP) regression and quadtree-based inference. LiDAR measurements are incrementally processed to refine poses and reconstruct continuous implicit surface representations, enabling accurate odometry and calibration in unstructured environments.

As illustrated in Figure 3, the GPIS-based odometry framework alternates between pose optimization and surface map updating. Let the environment be modeled as an SDF f(x), and let the transformation T=[R∣t] align the observed point $x_{i}$ with the surface where f(x)=0. The optimization objective is to minimize the distance to the surface:

(1) $$T=\arg\min_{R,t}\sum_{i}{∥f\left(Rx_{i}+t\right)∥}^{2}$$

This optimization problem is solved iteratively as follows:

1. Initialize the pose $T_{\mathrm{init}}$.
2. Transform $x_{i}$ using $T_{\mathrm{init}}$.
3. Solve for $T_{*}$ using the closed-form solution in Equation (A7).
4. If $∥T_{*}∥<\epsilon$, stop; otherwise, update $T_{\mathrm{init}}\leftarrow T_{\mathrm{init}}\cdot \exp\left(T_{*}\right)$ and repeat.

The transformation is refined by solving a confidence-weighted linear system derived from the GPIS gradient field and spatial Jacobians. This approach enables robust incremental motion estimation without requiring explicit feature correspondences or loop closure. The full mathematical derivation and weight definition are provided in Appendix A.

#### 3.2.2. Map Updating

We adopt an online map updating strategy based on GPIS [31]. This method allows for continuous, accurate environmental mapping from sparse LiDAR observations while preserving essential structural features. It incorporates the following three modules:

- GP regression and map update,
- inference of map surface points,
- computational acceleration techniques.

These integrated modules ensure both efficiency and real-time capability during GPIS-based mapping.

**Figure 4:**
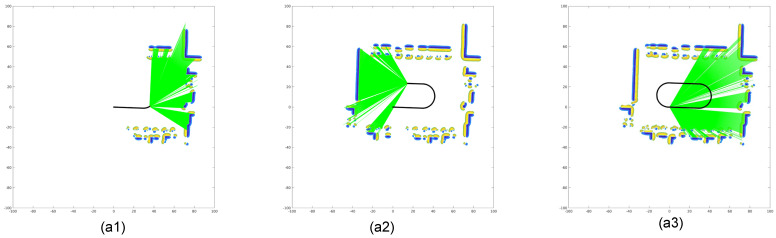

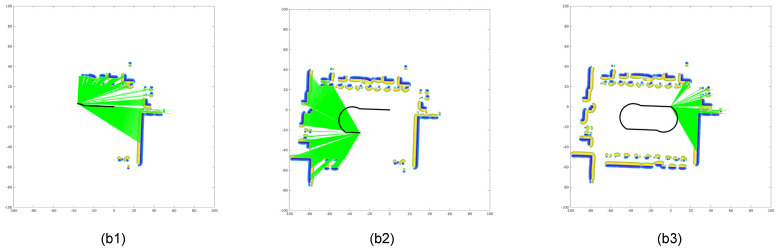
Two-dimensional LiDAR odometry using GPIS. (**a1**–**a3**) Consecutive reference LiDAR frames showing ray projections and the estimated trajectory. (**b1**–**b3**) Corresponding target LiDAR frames processed with the GPIS-based odometry. Yellow and blue regions represent positive and negative values of the SDF, respectively.

Figure 4 presents the odometry results from both reference and target LiDAR frames. The black trajectory shows the estimated self-motion, while the green rays represent current LiDAR observations. The GPIS map is constructed by sampling the SDF. Positive SDF values (yellow) correspond to external surfaces, while negative values (blue) indicate internal structures. In regions with high variance, the confidence scores are reduced, and the corresponding map points are discarded. Notably, even in the absence of loop closure, the trajectory demonstrates excellent internal consistency, with no observable drift or ghosting upon returning to the origin.

**Figure 5:**
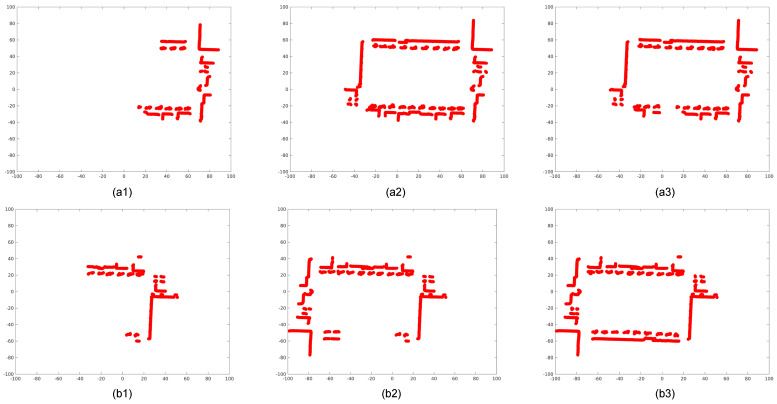
Progressive reconstruction of zero-level surfaces in the GPIS map. (**a1**–**a3**) Zero-level surfaces inferred from three consecutive reference LiDAR frames. (**b1**–**b3**) Zero-level surfaces inferred from the corresponding target LiDAR frames. The red points highlight the extracted GPIS surface points, illustrating temporal consistency and structural continuity across frames.

To further illustrate the spatial consistency of surface point reconstruction during odometry, Figure 5 presents the evolution of zero-level surface points extracted across multiple time steps. It demonstrates how GPIS-based map inference progressively reconstructs structured environmental geometry without relying on explicit feature correspondences.

### 3.3. Extrinsic Calibration via Motion and Surface Alignment

Level set methods [38] offer a powerful framework for evolving surface geometries, which is particularly beneficial when dealing with implicit models. Given that the GPIS maps from the reference and target LiDARs exhibit partial consistency within their respective odometry frames, we perform a two-stage optimization process as follows:

#### 3.3.1. Initial Value Estimation via Relative Motion

**Figure 6:**
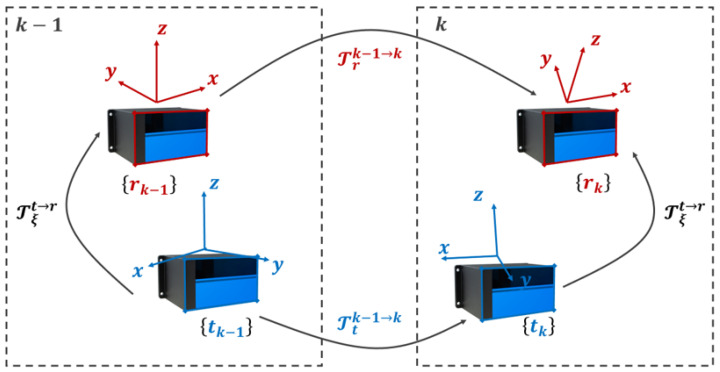
Relative motion model between LiDAR sensors. The schematic illustrates the temporal relationship between the reference and target LiDAR ego-motions and the fixed extrinsic transformation $T_{t\to r}^{\xi }$. This formulation supports a hand–eye calibration structure that does not rely on overlapping fields of view or artificial calibration targets.

In this first stage, we estimate the initial transformation between the non-overlapping LiDARs based on relative motion constraints. This method does not require overlapping fields of view or artificial targets, making it ideal for scenarios with limited or no overlap between sensor fields. Figure 6 shows the constrained relative motion between sensors for decoupled optimization.

Let the poses of the reference and target LiDARs at times k−1 and *k* be denoted as $T_{r_{k-1}},\;T_{r_{k}},\;T_{t_{k-1}},\;T_{t_{k}}$, respectively. The extrinsic transformation $T_{\xi }^{t\to r}$ remains constant across time and satisfies the following relationships:

(2) $$\begin{array}{cc} T_{r_{k}} & =T_{r}^{k-1\to k}\;T_{r_{k-1}},\\ T_{t_{k}} & =T_{t}^{k-1\to k}\;T_{t_{k-1}},\\ T_{r_{k-1}} & =T_{\xi }^{t\to r}\;T_{t_{k-1}},\\ T_{r_{k}} & =T_{\xi }^{t\to r}\;T_{t_{k}}. \end{array}$$

These transformation relationships, shown in Equation (2), link the consecutive poses of the two LiDARs through their ego-motions and a fixed extrinsic calibration.

By eliminating the absolute poses from the above formulation, we arrive at the classic hand–eye calibration form:

(3) AX=XB

where

$$A=T_{r}^{k-1\to k},\;B=T_{t}^{k-1\to k},\;X=T_{\xi }^{t\to r}.$$

Instead of using a closed-form decomposition, we formulate the calibration as a non-linear optimization problem:

(4) $$\min_{\xi }\sum_{k=1}^{N}w_{k}\;G\left({\left(T_{\xi }^{-1}T_{r}^{k-1\to k}T_{\xi }\right)}^{-1}T_{t}^{k-1\to k}\right)$$

where G(·) denotes a residual function that measures the discrepancy between the aligned transformations, and $w_{k}$ is a confidence weight for each motion pair.

This optimization is solved using the Ceres Solver (version 2.1.0), which provides robust and fast convergence of the extrinsic parameter $T_{\xi }^{t\to r}$.

#### 3.3.2. Refinement via ICP on GPIS Surface Points

**Figure 7:**
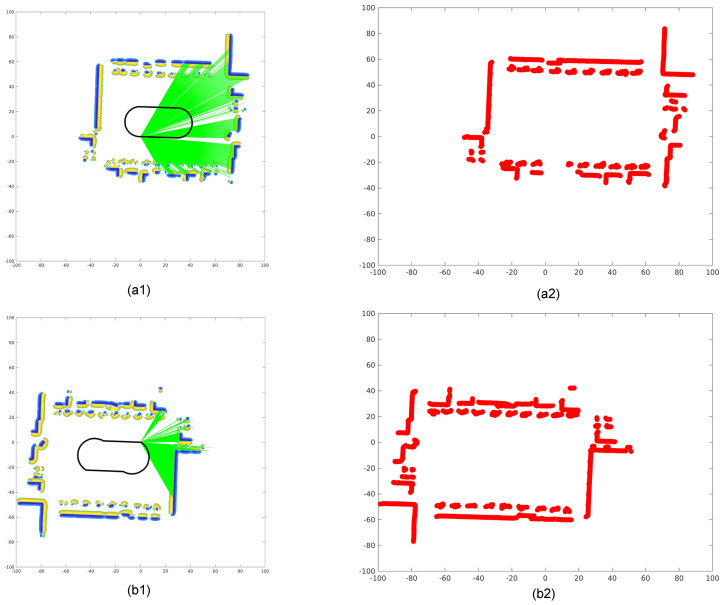
Surface point extraction from GPIS maps. Panels (**a1**,**b1**) show the continuous implicit surface maps generated from reference and target LiDAR odometries. Panels (**a2**,**b2**) visualize the extracted zero-level surface points used for subsequent ICP alignment. The smooth and noise-suppressed GPIS surfaces provide a reliable geometric basis for extrinsic refinement.

Once the GPIS maps are generated from both the reference and target LiDAR odometries, we extract environmental surface points using zero-level sets of the SDF, as depicted in Figure 7.

**Figure 8:**
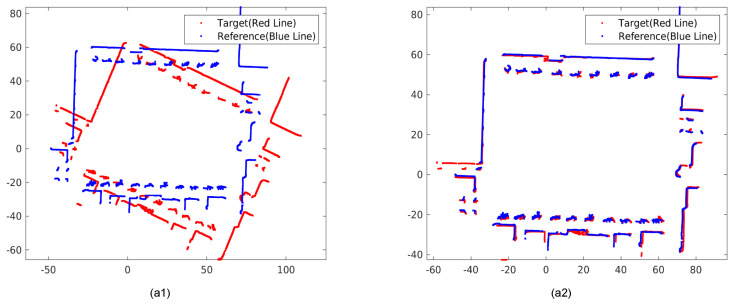
ICP-based refinement of extrinsic parameters. Panel (**a1**) shows the initial extrinsic misalignment with a yaw offset of 158°. Panel (**a2**) shows the result after applying ICP on the GPIS-derived zero-level surfaces. Despite the large initial rotation error, the optimization converges accurately, which validates the robustness of the surface-level alignment.

To refine the initial extrinsic parameters, we apply the ICP algorithm to these extracted surface points. The alignment results before and after ICP optimization are shown in Figure 8. Despite large initial angular offsets, such as 158°, the ICP process consistently converges to an accurate registration. Higher precision is subsequently achieved by optimizing over the GPIS-defined surfaces. These surfaces provide a continuous and noise-suppressed representation, which contributes to the accuracy of the optimization process.

### 3.4. Implementation Details

#### 3.4.1. GPIS Kernel Configuration

We employ a 2D thin-plate spline (TPS) covariance function for GPIS modeling:

(5) $$k\left(x_{i},x_{j}\right)=\sigma_{f}^{2}\;r^{2}\log\left(r+\epsilon \right)+\sigma_{n}^{2}\delta_{ij},$$

where $r=∥x_{i}-x_{j}{∥}_{2}$ is the Euclidean distance, ϵ is a small constant for numerical stability when r→0, $\sigma_{f}=1.0$ is the signal variance, and $\sigma_{n}=0.1$ is the noise variance (nugget term). Unless otherwise stated, these hyperparameters are kept fixed across all experiments for reproducibility.

#### 3.4.2. Optimization Settings

Motion-based initialization is solved using the Ceres Solver with the Levenberg–Marquardt algorithm, a trust-region radius of $10^{-4}$, and convergence tolerances of $10^{-6}$ for both the cost function and parameter updates. ICP refinement employs point-to-plane minimization with an outlier rejection threshold of 0.1 m and a maximum of 50 iterations.

#### 3.4.3. Control Point Sampling

For backward–forward projection evaluation, we uniformly sample 40,000 control points from the reference LiDAR frame. The GPIS map is updated at 1 Hz with a spatial resolution of 0.5 m using a quadtree structure.

## 4. Results

### 4.1. Experiments on Prescan

To validate the effectiveness and robustness of the proposed non-overlapping LiDAR calibration framework, we conducted extensive experiments in a simulated environment using PreScan (version 2021.1). This section outlines the experimental setup, compares calibration accuracy with multiple baseline methods, and evaluates robustness under varying levels of Gaussian noise. We adopt evaluation metrics consistent with recent benchmarks [6], including translation error ∥δr∥ and angular error ∥δθ∥.

#### 4.1.1. Simulation Environment and Dataset

**Figure 9:**
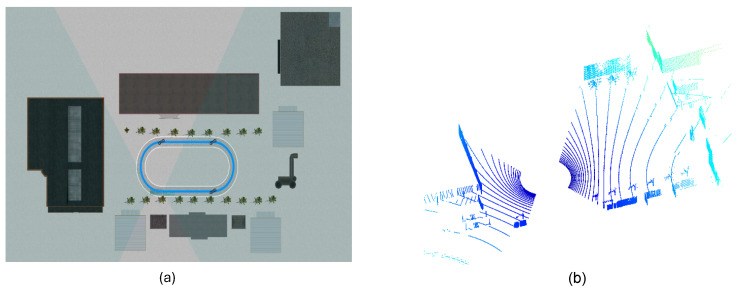
PreScan simulation platform for multi-LiDAR calibration. (**a**) Interface of the PreScan scenario editor. (**b**) Simulated LiDAR point cloud outputs under non-overlapping field-of-view (FoV) configurations. This environment enables controlled evaluation of calibration performance under varying noise levels and sensor layout conditions.

All experiments were carried out in the high-fidelity PreScan simulation platform [39,40], which offers realistic modeling of LiDAR sensing physics and noise. The scene includes multiple LiDAR sensors mounted with minimal field-of-view (FoV) overlap, reflecting typical deployment challenges in real-world vehicle systems. Figure 9 illustrates the simulated multi-LiDAR configuration used for validation.

Each LiDAR sensor uses 32 vertical channels with a scanning range of +6° to −4°, consistent with the specifications of the Leishen CH32 solid-state LiDAR. Ground-truth extrinsic parameters are available, enabling precise quantitative evaluation of our calibration method.

#### 4.1.2. Horizontal Extrinsic Calibration Accuracy

We evaluate the horizontal components (x,y,θ) of the estimated extrinsic parameters by comparing them to the ground truth. Here, (x,y) represent lateral displacements in meters and θ is the yaw angle in degrees. The planar error magnitude is computed as $∥\delta_{r}∥=\sqrt{\delta_{x}^{2}+\delta_{y}^{2}}$.

Baseline Methods. *3D-IO-M* [41] estimates LiDAR poses through pairwise 3D ICP between successive scans to build dense point clouds for alignment. *ALOAM-M*, based on the LOAM algorithm [42], performs LiDAR odometry with optimized feature selection, followed by map-based calibration. *2D-NO-M* leverages Normal Distributions Transform (NDT) [16] in a 2D BEV space for odometry estimation, using map alignment to estimate extrinsics.

Ours. Our approach combines relative motion-based estimation with ICP refinement on GPIS zero-level surfaces. As shown in Table 2, our method outperforms all baselines, achieving a horizontal translation error of 7 cm and angular error of 0.158°, demonstrating superior accuracy in non-overlapping configurations.

#### 4.1.3. Projection Error Visualization and Analysis

To quantitatively evaluate calibration accuracy, we adopt the backward–forward projection error as a geometric consistency metric.

**Figure 10:**
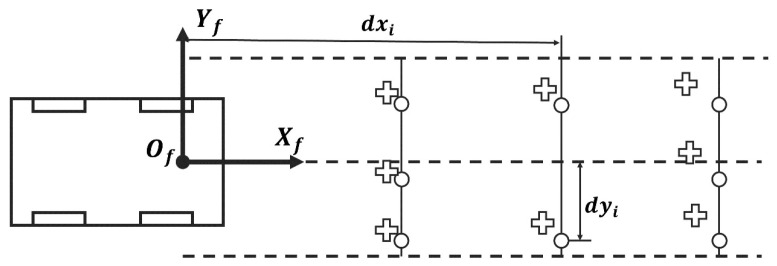
Schematic of the backward–forward projection process. Control points (circles) are sampled from the reference LiDAR frame and projected into the target frame using ground-truth extrinsic parameters. The projected points are then reprojected back using the estimated parameters, yielding reprojected points (crosses). The Euclidean distance between the original and reprojected points defines the backward–forward projection error metric.

As shown in Figure 10, control points are sampled from the reference LiDAR frame (denoted by circles). These points are projected into the target frame using ground-truth extrinsics, generating the backward-projected points. These points are then reprojected back into the reference frame using the estimated extrinsics, resulting in the reprojected points (crosses). The discrepancy between the original and reprojected points represents the error introduced by the extrinsic estimation.

The backward–forward projection error $e_{\mathrm{BF}}$ is defined as the average Euclidean distance between each original point $p_{i}$ and its reprojected counterpart $p_{i}^{\prime }$, formulated as:

(6) $$e_{\mathrm{BF}}=\frac{1}{N}\sum_{i=1}^{N}{∥p_{i}-p_{i}^{\prime }∥}_{2}$$

We evaluate the distribution of this error by randomly sampling 40,000 control points from the reference frame and visualizing the results in both 3D and projected 2D space.

**Figure 11:**
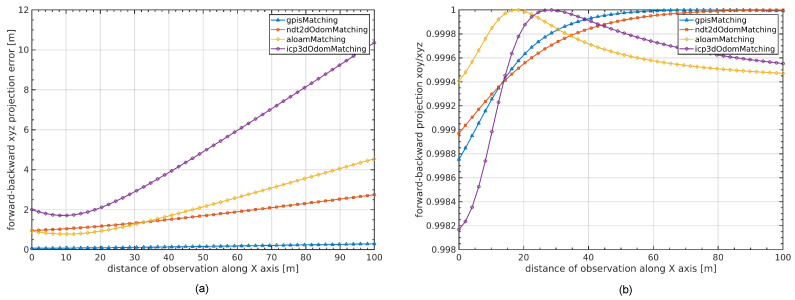
Backward–forward projection analysis. (**a**) Three-dimensional backward–forward projection error $e_{\mathrm{BF}}^{\mathrm{xyz}}$, quantifying cumulative spatial drift along the observation trajectory. (**b**) Ratio between two-dimensional ($e_{\mathrm{BF}}^{\mathrm{xy}}$) and three-dimensional ($e_{\mathrm{BF}}^{\mathrm{xyz}}$) projection errors, highlighting angular misalignment. The four compared methods include *gpisMatching* (proposed), *ndt2dOdomMatching* (2D-NO-M), *aloamMatching* (ALOAM-M), and *icp3dOdomMatching* (3D-IO-M). Across all distances, the proposed method consistently achieves the lowest projection error, indicating improved calibration stability at long ranges.

As shown in Figure 11a, baseline methods such as 3D-IO-M exhibit significant drift due to angular errors, while our method remains highly consistent, maintaining sub-meter accuracy across the entire range. Figure 11b shows the ratio between horizontal (XOY) and full-space projection errors. Our method remains close to 1.0, indicating accurate alignment in all spatial axes.

**Figure 12:**
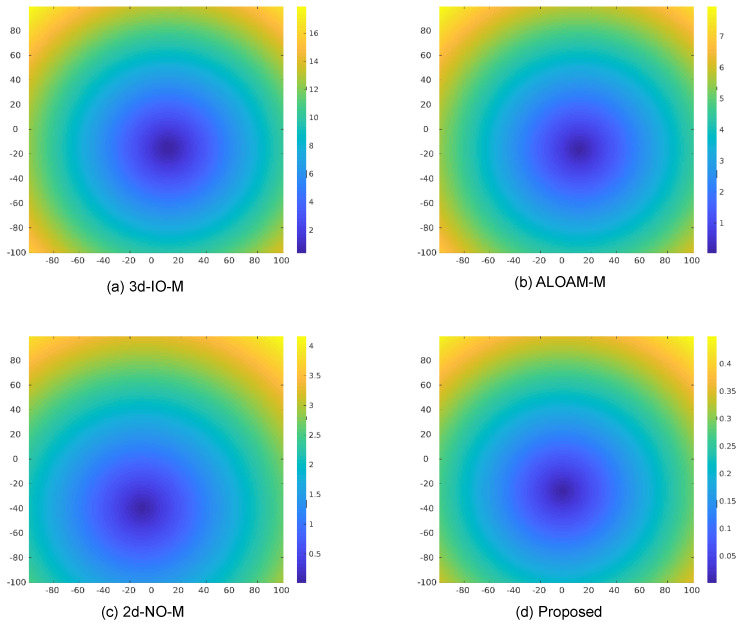
Top-view backward–forward projection error map in the xoy plane at a 100 m observation range. Panels show the two-dimensional backward–forward projection error $e_{\mathrm{BF}}^{xoy}$ for four calibration methods: (**a**) 3D-IO-M, (**b**) ALOAM-M, (**c**) 2D-NO-M, and (**d**) the proposed GPIS-based method. Warmer colors indicate larger projection errors. Among the evaluated approaches, the proposed method yields the lowest error (0.4 m), whereas 3D-IO-M exhibits the highest (16 m), illustrating the impact of accurate extrinsic calibration.

As shown in Figure 12, we visualize the 2D backward–forward projection error $e_{\mathrm{BF}}^{xoy}$ for different calibration methods at a 100-meter observation distance. Compared to the baseline methods—3D-IO-M (16m), ALOAM-M (7m), and 2D-NO-M (4m)—our GPIS-based approach significantly reduces the projection error to only 0.4m, demonstrating superior angular alignment and robustness in long-range scenarios.

#### 4.1.4. Robustness Under Gaussian Noise

To evaluate the robustness of our method under sensor noise, we inject Gaussian noise with varying standard deviations ($\sigma_{r}$) into the LiDAR range data and compute the average backward–forward projection error.

As shown in Table 3, our method demonstrates superior resilience to noise, maintaining low projection errors across all tested noise levels. In contrast, 3D-IO-M and ALOAM-M show significant degradation at higher noise levels, validating that our GPIS-based approach effectively mitigates the impact of noisy observations.

### 4.2. Preliminary Real-Data Validation on nuScenes

#### 4.2.1. Experimental Setup

To partially address the lack of real-world validation, we conduct a preliminary case study on the nuScenes dataset. Since nuScenes provides only a single physical LiDAR sensor, we emulate a realistic non-overlapping dual-LiDAR configuration by splitting each scan into front-view and rear-view subsets according to the sensor heading. This setup mimics two LiDARs mounted with opposite viewing directions and non-overlapping fields of view, which is a challenging scenario for calibration methods relying on direct scan overlap. Under this construction, the ground-truth relative extrinsic between the front and rear subsets is known and fixed as $\left(x,y,\mathrm{yaw}\right)=\left(0,0,180^{{}^{\circ}}\right)$. This allows us to quantitatively evaluate calibration accuracy by measuring absolute parameter errors. We perform 2.5D extrinsic calibration over an entire driving sequence (whole-trajectory estimation), estimating only horizontal parameters (x,y,yaw). Vertical parameters are intentionally excluded, consistent with the engineering-oriented design of the proposed method.

#### 4.2.2. Baselines and Evaluation Metrics

We compare the proposed GPIS-based method with several representative baselines: (i) a motion-only hand–eye calibration method based on ego-motion estimates and (ii) BEV-based registration methods, including 2D ICP and 2D NDT. All methods are evaluated using absolute parameter errors, including |Δx|, |Δy|, and absolute angular error |Δyaw| after angle wrapping. Note that yaw accuracy is evaluated as deviation from the known ground truth of 180°. Due to the limited revision period, results are reported on one representative nuScenes sequence. The purpose of this experiment is to demonstrate feasibility and relative performance trends on real sensor data, rather than to provide exhaustive statistical evaluation.

#### 4.2.3. Quantitative Results

We conduct two real-data experiments on the nuScenes dataset to analyze the behavior of different calibration strategies under different prior conditions. Both experiments are performed under a non-overlapping dual-LiDAR configuration constructed by front/rear splitting, and all results are reported as absolute parameter errors with respect to the ground truth $\left(x,y,\mathrm{yaw}\right)=\left(0,0,180^{{}^{\circ}}\right)$.

##### Experiment I: Calibration Without GPS Prior

Experiment I evaluates calibration performance when no global positioning prior is available. In this setting, the motion-based hand–eye baseline relies solely on ego-motion estimated via 3D ICP, while BEV-based ICP and NDT operate on 2D projections. This experiment is not intended to demonstrate superiority among different methods. Instead, it highlights the inherent difficulty of non-overlapping extrinsic calibration when no reliable global prior is available. As shown in Table 4, all methods exhibit large translational and angular errors. In particular, BEV-based methods suffer from severe performance degradation due to the loss of geometric constraints in road-dominant environments, while the proposed method behaves similarly to motion-based hand–eye calibration under weak prior conditions.

##### Experiment II: Calibration with GPS Prior

In Experiment II, a coarse GPS prior is provided as initialization. With the availability of global positioning information, overall trajectory alignment errors are significantly reduced for all methods, and iterative optimization converges more rapidly.

As reported in Table 5, the proposed GPIS-based method achieves smaller yaw error and competitive lateral accuracy compared to motion-based hand–eye calibration, benefiting from environment-level surface constraints. These results demonstrate the effectiveness of the proposed approach as a refinement stage. rather than a standalone initializer, which is consistent with its intended engineering-oriented design.

#### 4.2.4. Observed Failure Cases and Discussion

We also observed failure cases in which the proposed pipeline does not return a valid solution. These cases typically occur when ego-motion excitation is insufficient (e.g., near-straight trajectories with limited rotation), when the scene is dominated by highly dynamic objects, or when the initialization from motion-only calibration is too far from the optimum. A systematic analysis of success rate across more sequences, as well as more robust initialization and failure recovery strategies, will be investigated in future work.

#### 4.2.5. Extension to Multi-Sensor Configurations

While our current experiments validate dual-LiDAR calibration, the framework is extensible to N≥3 sensors through global consistency constraints. For three or more LiDARs, we recommend a hierarchical approach: first establish pairwise calibrations between adjacent sensors, then enforce global consistency through bundle adjustment over all sensor pairs. This mitigates pairwise error accumulation by jointly optimizing all extrinsic parameters with temporal constraints. Synchronization issues can be addressed by incorporating time offset estimation in the optimization, while differing scan rates may require temporal interpolation of point clouds. Future work will include experimental validation on three-LiDAR setups to demonstrate these extensions.

### 4.3. Computational Cost and Scalability

We evaluated the computational performance of our GPIS-based calibration framework on a desktop workstation (Intel Core i7-8700K CPU, Intel Corporation, Santa Clara, CA, USA; 32 GB RAM; NVIDIA RTX 3080 GPU, NVIDIA Corporation, Santa Clara, CA, USA). Table 6 reports the per-stage runtime and peak memory footprint on a representative sequence of 1000 frames with approximately 50,000 points per scan.

The scalability terms follow Big-O notation, where *N* is the number of raw points per scan, *M* is the number of GPIS support/map elements involved in regression and inference, *T* is the temporal window length used for motion constraints, and *K* is the number of ICP iterations. Overall, the pipeline runs at approximately 3.3 Hz on our test sequence. GPIS odometry and the initialization stage dominate runtime; memory usage is mainly driven by GPIS map maintenance and support-point storage. Improving real-time capability via sparse/incremental GPIS updates is an important direction for future work.

## 5. Discussion

The experimental results in the PreScan environment demonstrate that the proposed GPIS-based framework can accurately calibrate non-overlapping dual-LiDAR systems and substantially outperforms representative baselines. From a sensor-system perspective, the framework provides an extrinsic calibration backend for dual-LiDAR suites on autonomous vehicles, enabling consistent geometric alignment under challenging deployment constraints. Compared with 3D-IO-M and ALOAM-M, which depend on explicit 3D feature correspondences and sufficient overlap, our method achieves centimeter-level horizontal translation accuracy and sub-degree yaw accuracy in minimally overlapping settings. The backward–forward projection analysis further shows that the drift of the proposed method remains below one meter at a 100 m range, whereas ICP-based approaches exhibit rapidly growing errors.

The main reason for this improvement lies in the implicit and probabilistic nature of GPIS modeling. Instead of directly aligning sparse and noisy point clouds, we construct a continuous implicit surface representation that encodes both geometric structure and uncertainty. This representation suppresses high-frequency noise, provides informative gradients for pose optimization, and yields a smoother objective for subsequent refinement. As a result, the calibration process becomes less sensitive to local outliers and missing measurements, which is crucial in unstructured scenes and practical non-overlapping dual-LiDAR configurations.

Compared with recent calibration systems such as Multi-LiCa [13] and integrated toolchains such as iKalibr [25], our approach targets the non-overlapping setting by introducing a GPIS-based implicit-surface layer that stabilizes map-level alignment without requiring full SLAM integration in the evaluated pipeline. Learning-based methods [43] are also promising, but they typically require substantial training data, and their generalization across sensor configurations remains an open challenge. Despite these advantages, the proposed framework has several limitations. First, the current implementation relies on batch optimization and GP regression, which constrains throughput when scaling to longer sequences or larger maps. Second, our primary evaluation is conducted in simulation; although the sensor models and noise statistics are realistic, real-world factors such as multi-path reflections, adverse weather, and mechanical deformation of sensor mounts are not yet captured. Future work will focus on improving efficiency via sparse/incremental GPIS updates, extending validation to real autonomous-vehicle multi-LiDAR rigs, and investigating global-consistency optimization for multi-sensor (N≥3) configurations.

## 6. Conclusions

This paper presents an engineering-oriented 2.5D extrinsic calibration framework for non-overlapping dual-LiDAR systems. By combining motion-guided planar alignment with GPIS-based implicit-surface constraints, the proposed method enables stable map-level alignment without requiring calibration targets or strong structural assumptions.

Extensive experiments in the PreScan environment demonstrate that our approach achieves centimeter-level horizontal translation accuracy and sub-degree yaw accuracy, consistently outperforming representative motion-/feature-based baselines under both clean and noisy settings. To further assess practical applicability, we additionally report a preliminary nuScenes case study using front/rear split point clouds to emulate a non-overlapping dual-LiDAR setup, where the proposed method yields improved yaw accuracy and competitive lateral precision.

Current limitations include the reliance on batch optimization and GP regression, which constrain throughput when scaling to longer sequences or larger maps, as well as the lack of full real-vehicle validation. Future work will investigate sparse/incremental GPIS updates to improve efficiency, validate the framework on real autonomous-vehicle multi-LiDAR rigs, and explore global-consistency optimization for multi-sensor (N≥3) configurations.

## Figures and Tables

**Table 1:** Representative multi-LiDAR calibration paradigms and typical limitations under non-overlapping setups.

Category	Common Constraint	Representation	Limitation Under Non-Overlap
Motion-based	Hand–eye/ego-motion	Trajectory	Sensitive to motion excitation; error accumulates with odometry drift
NDT/BEV-based	Map-level structure	BEV/NDT grid	Requires structured scenes; quantization and local minima
LOAM/feature-SLAM style	Map-level feature overlap	Sparse features	Feature sparsity in weak structure; drift accumulation
ICP/dense registration	Map-level dense overlap	Dense point map	Correspondences unstable under noise/outliers; higher runtime
Target-based	Artificial targets	Target correspondences	Requires target deployment and maintenance
Ours (GPIS-aided)	Map-level surface consistency	GPIS implicit surface	Adds a GPIS layer to denoise and stabilize optimization (2.5D evaluated)

**Table 2:** Extrinsic horizontal component estimation errors ($∥\delta_{r}∥=\sqrt{\delta_{x}^{2}+\delta_{y}^{2}}$).

Method	(x,y,θ) [m, deg]	$∥\delta_{x}∥$	$∥\delta_{y}∥$	$∥\delta_{\theta }∥$	$∥\delta_{r}∥$
Ground Truth	(−7.354,0.247,−179.912)	0	0	0	0
3D-IO-M	(−5.568,−1.532,−173.365)	1.786	1.779	6.546	2.521
ALOAM-M	(−6.567,−0.597,−177.047)	0.787	0.844	2.864	1.154
2D-NO-M	(−6.437,0.343,−178.574)	0.917	0.095	1.338	0.922
**Ours**	** (−7.284,0.235,−179.753) **	**0.069**	**0.011**	**0.158**	**0.070**

**Table 3:** Average backward–forward projection error under Gaussian noise (${\bar{e}}_{\mathrm{BF}}^{xyz}$, in m).

Noise Std. Dev. ($\sigma_{r}$)	3D-IO-M	ALOAM-M	2D-NO-M	Ours
0.00	8.875	3.907	1.966	**0.221**
0.05	7.933	4.249	1.547	**0.316**
0.08	9.094	4.526	1.199	**0.328**
0.10	8.240	4.981	1.944	**0.333**
0.20	11.062	20.989	1.106	**0.380**

**Table 4:** Calibration results on one representative nuScenes sequence without GPS prior. This experiment highlights the limitations of ego-motion-based initialization under non-overlapping conditions. All values are reported as absolute errors.

Method	Method Assumptions			Absolute Calibration Error		
	Uses Odom	Uses Env	Non-Overlap	|Δx| (m)	|Δy| (m)	|Δyaw| (deg)
Motion-based Hand–Eye (3D ICP)	✓	✓	✓	0.90	2.40	6.3
2D ICP (BEV)	✓	✓	✓	1.20	3.00	7.8
2D NDT (BEV)	✓	✓	✓	1.41	2.86	8.5
**Ours (GPIS-based 2.5D)**	✓	✓	✓	**0.83**	**1.77**	**7.1**

**Table 5:** Calibration results on one representative nuScenes sequence with GPS prior. All values are reported as absolute errors.

Method	GPS Prior	Uses Odom	Uses Env	Non-Overlap	Absolute Calibration Error		
					|Δx| (m)	|Δy| (m)	|Δyaw| (deg)
Motion-based Hand–Eye (3D ICP)	✓	✓	✓	✓	0.21	0.37	1.3
2D ICP (BEV)	✓	✓	✓	✓	0.40	0.74	4.1
2D NDT (BEV)	✓	✓	✓	✓	0.55	0.79	2.3
**Ours (GPIS-based 2.5D)**	✓	✓	✓	✓	**0.16**	**0.40**	**0.7**

**Table 6:** Computational cost breakdown for the GPIS-based calibration pipeline.

Stage	Runtime (ms)	Peak Memory (MB)	Asymptotic Scaling
Preprocessing (ground estimation + BEV)	25.3	120	O(N)
GPIS odometry (per LiDAR)	45.7	850	O(MlogM)
Hand–eye initialization	120.5	200	O(T)
GPIS surface inference	35.2	600	O(M)
ICP refinement (default library implementation)	78.9	150	O(KMlogM)
**Total (per frame)**	**305.6**	**1920**	–

## Data Availability

The simulation scenarios and calibration scripts used in this study are openly available on Zenodo at https://doi.org/10.5281/zenodo.17693506 (accessed on 25 November 2025).

## Appendix A. Derivation of the GPIS-Based Pose Estimation

This appendix provides the detailed derivation of the pose estimation process used in GPIS-based LiDAR odometry.

Given a new LiDAR frame, the pose is estimated by minimizing the distance between the transformed points and the zero-level set of the SDF, defined as:

(A1) $$T=\arg\min_{R,t}\sum_{i}{∥f(Rx_{i}+t)∥}^{2}$$

Using the small-angle approximation $R\approx {\left[\omega \right]}_{\times }+I$, the displacement of each point can be approximated as:

(A2) $$\delta x_{i}\approx {\left[\omega \right]}_{\times }x_{i}+t$$

Applying a first-order Taylor expansion of the SDF yields:

(A3) $$f\left(x_{i}+\delta x_{i}\right)\approx f\left(x_{i}\right)+\nabla f_{x_{i}}^{T}\delta x_{i}$$

The expression can be linearized as:

(A4) $$f\left(x_{i}+\delta x_{i}\right)\approx f\left(x_{i}\right)+\left[\begin{array}{cc} \nabla f_{x_{i}}^{T} & {\left(\nabla f_{x_{i}}\times x_{i}\right)}^{T} \end{array}\right]\left[\begin{array}{c} t\\ \omega \end{array}\right]$$

Letting

$$b_{i}=\left[\begin{array}{c} \nabla f_{x_{i}}\\ \nabla f_{x_{i}}\times x_{i} \end{array}\right],\;T_{*}=\left[\begin{array}{c} t\\ \omega \end{array}\right],$$

the loss function becomes:

(A5) $$L\left(T_{*}\right)=\sum_{i}\left(2f\left(x_{i}\right)b_{i}^{T}T_{*}+T_{*}^{T}b_{i}b_{i}^{T}T_{*}\right)$$

By setting the gradient to zero, i.e., $\frac{\partial L}{\partial T_{*}}=0$, we derive the closed-form solution:

(A6) $$T_{*}=-{\left(\sum_{i}b_{i}b_{i}^{T}\right)}^{-1}\left(\sum_{i}f\left(x_{i}\right)b_{i}\right)$$

To incorporate measurement uncertainty, we introduce confidence weights $w({\mathrm{cov}}_{i})$ and obtain the weighted form:

(A7) $$\begin{array}{cc} T_{*}= & -{\left(\sum_{i}w\left({\mathrm{cov}}_{i}\right)\;b_{i}b_{i}^{T}\right)}^{-1}\\ \; & \cdot \left(\sum_{i}w\left({\mathrm{cov}}_{i}\right)f\left(x_{i}\right)\;b_{i}\right) \end{array}$$

where the confidence weight function is defined as:

(A8) $$w\left({\mathrm{cov}}_{i}\right)=2-\frac{2}{1+e^{-{\mathrm{cov}}_{i}}}$$

The iterative optimization process follows the standard procedure described in Section 3.2: initialize the pose, apply the transformation, solve for the optimal update, and repeat until convergence. This appendix focuses on the mathematical derivation of the update step summarized in Equation (A7).

This strategy follows from GPIS-based surface modeling and confidence-weighted inference techniques [31,44].
